# Differential Impact of the Endometrial Immune Environment in Fresh Versus Frozen Embryo Transfer

**DOI:** 10.3390/cells15131186

**Published:** 2026-06-30

**Authors:** Nathalie Lédée, Nada J. Habeichi, Mona Rahmati, Géraldine Dray, Nicole Kerkhoven, Eric Vicaut, Abdourahmane Diallo, Nino Guy Cassuto, Lea Ruoso, Laura Prat-Ellenberg, Marie Petitbarat

**Affiliations:** 1 Centre d’Assistance Médicale à la Procréation, Hôpital des Bluets, 4 Rue Lasson, 75012 Paris, France; geraldine.dray@bluets.org (G.D.); laura.prat-ellenberg@bluets.org (L.P.-E.); 2 MatriceLab Innove Laboratory, Immeuble Les Gemeaux, 2 Rue Antoine Etex, 94000 Créteil, France; nada.habeichi@matricelabinnove.com (N.J.H.); nicole.kerkhoven@matricelabinnove.com (N.K.); marie.petitbarat@matricelabinnove.com (M.P.); 3 UFR Simone Veil Santé, UVSQ, Université Paris Saclay, 78180 Montigny le Bretonneux, France; 4 London Women’s Clinic, London W1G 6AP, UK; mona.rahmati@londonwomensclinic.com; 5 Unité de Recherche Clinique, Hôpital Fernand Widal, APHP, Université Paris-Diderot Paris 7, 75010 Paris, France; eric.vicaut@aphp.fr (E.V.); abdourahmane.diallo@aphp.fr (A.D.); 6 Laboratoire Drouot, 21 Rue Drouot, 75010 Paris, France; guycassuto@labodrouot.com (N.G.C.); learuoso@labodrouot.com (L.R.)

**Keywords:** endometrial immune profiling, uterine immune environment, precision care, IVF, frozen embryo transfer (FET), live birth rate (LBR), cytotoxicity at MFI, trophoblast invasion, spiral artery remodeling, tolerogenic molecules

## Abstract

**Highlights:**

**What are the main findings?**

- Endometrial immune status had a markedly stronger impact on implantation outcomes in frozen compared with fresh embryo transfer cycles.
- Immune-guided precision care significantly improved live birth rates in patients with endometrial immune dysregulation undergoing frozen embryo transfer.

**What are the implications of the main findings?**

- The influence of the uterine immune environment on implantation appears to depend on both the hormonal environment and whether embryos are transferred fresh or after cryopreservation.
- Endometrial immune profiling may help identify patients at higher risk of implantation failure during frozen embryo transfer and support more personalized IVF treatment strategies.

**Abstract:**

Background: The endometrial immune environment regulates maternal immune tolerance and plays a key role in embryo implantation. We investigated whether its impact on reproductive outcomes differs between fresh and frozen embryo transfer (FET) cycles. Methods: In this prespecified secondary analysis of a prospective randomized study, 493 good-prognosis IVF patients underwent endometrial immune profiling before embryo transfer. Patients with a balanced profile received standard care, whereas those with immune dysregulation were randomized to conventional or immune-guided precision care. Live birth rates (LBRs) were analyzed according to immune status, transfer type, and treatment strategy. Results: A transfer type-specific effect was observed. In FET cycles, a balanced immune profile was associated with higher LBR compared with immune dysregulation (52.4% vs. 14.7%; adjusted OR 5.32 [95% CI 1.53–20.92]; ARD +37.7%). Precision care improved LBR compared with conventional management (48.6% vs. 14.7%; OR 5.03 [1.66–17.63]; ARD +33.9%). No significant association between immune status and reproductive outcomes was observed in fresh transfers. Conclusions: The impact of the uterine immune environment differed according to embryo transfer type and was predominantly observed in FET cycles. These findings suggest that endometrial immune competence may be particularly relevant to implantation success after frozen embryo transfer and warrants confirmation in larger prospective studies.

## 1. Introduction

Assisted reproductive technologies (ART) have markedly expanded access to infertility treatment, yet implantation failure remains a major limitation to overall success [1]. Even among women younger than 35 years, live birth rates (LBR) per initiated IVF cycle remain close to 30%, with outcomes declining progressively with maternal age [2]. Beyond biological constraints, repeated treatment failure generates considerable emotional distress and imposes substantial psychological, social, and economic burdens on affected couples [3].

During the past decade, frozen–thawed embryo transfer (FET) has progressively become an integral component of IVF practice. Improvements in embryo vitrification, embryo culture systems, and the introduction of blastocyst biopsy for preimplantation genetic testing have contributed to the rapid expansion of FET worldwide [4]. FET has also emerged as a safer treatment option because it significantly reduces the risk of ovarian hyperstimulation syndrome (OHSS), one of the major complications associated with assisted reproductive technologies. Consequently, frozen embryo transfer is increasingly used not only as a complementary strategy but in some cases as an elective alternative to fresh embryo transfer. In 2018, FET accounted for approximately 65% of all IVF cycles in Europe, while fresh embryo transfers accounted for roughly 35% [2]. This trend is not restricted to Europe and has also been observed across Asian IVF registries, reflecting the worldwide adoption of cryopreservation-based embryo transfer strategies [5,6].

One of the proposed advantages of FET is that embryo transfer can be performed in a uterine environment that more closely resembles physiological conditions [7]. In contrast, fresh embryo transfers occur shortly after ovarian stimulation, when supraphysiological concentrations of ovarian steroids alter endometrial receptivity and disrupt embryo–endometrium synchrony [8]. Accumulating evidence indicates that ovarian hyperstimulation disrupts endometrial decidualization and alters immune signaling pathways [9,10,11]. Although freeze-all strategies (elective cryopreservation of all embryos for later transfer) are clearly beneficial in selected clinical situations [12,13], the decision to perform fresh or frozen embryo transfer in routine practice remains largely empirical and is frequently driven by embryo-related considerations rather than endometrial factors [14,15].

Successful implantation relies on a tightly regulated dialogue between the embryo and the maternal endometrium during the window of implantation in mid luteal phase, when decidualization establishes a transient receptive state associated with extensive remodeling of the local immune environment. Endometrial innate immune cells, particularly uterine natural killer (uNK) cells, together with cytokine networks, orchestrate trophoblast invasion, vascular remodeling, and early placental development [16,17,18]. During this period, the immune milieu shifts towards innate, tolerance-promoting mechanisms, in which a balanced Th1/Th2 cytokine profile is critical, as an excessive inflammatory Th1 immune activation may impair implantation whereas a modulatory Th2 dominant signaling would support immune adaptation and tissue remodeling [19,20,21,22].

Within this framework, key immune pathways involving Interleukin-18 (IL-18), Interleukin-15) IL-15, TWEAK (Tumor necrosis factor-like weak inducer of apoptosis), Fn-14 (Fibroblast Growth Factor-Inducible 14) were identified through a translational approach combining abortive murine models and integrative human studies, including immunohistochemical, transcriptomic, and functional microhistoculture analyses [23]. Quantification of these biomarkers defines the endometrial immune profile during the mid-luteal phase and identifies dysregulation associated with implantation failure.

The IL-18/TWEAK mRNA ratio reflects angiogenesis and immunoregulated Th1/Th2 balance, whereas IL-15/Fn-14 assesses uNK cell activation and maturation, in addition to CD56 assessment, estimating their recruitment. The uterine immune profile translates complex immune mechanisms underlying implantation into a clinically actionable framework, with the aim of guiding precision medicine through integration of key local immune responses [24]. When performed during the mid-luteal phase, this assessment allows classification of the endometrial immune environment as balanced or dysregulated [23].

As previously stated, the endometrial immune profile does not necessarily reflect a pathological uterine condition, but may instead indicate a less receptive immune environment for implantation. This “less receptive environment” refers to insufficient uterine immune preparation, which may hinder successful embryo attachment and implantation if the embryo is unable to compensate for the local imbalance [25]. In a recent randomized controlled trial based on the same cohort, we demonstrated that precision care guided by endometrial immune profiling improved live birth rates in good-prognosis IVF patients with immune dysregulation, compared with conventional management [25].

The present study is a pre-specified secondary analysis designed to address a distinct question: whether the impact of the endometrial immune environment differs between fresh and frozen embryo transfer cycles. As fresh and frozen transfers occur in different endocrine contexts, the impact of local immune balance on implantation may also vary with the transfer strategy.

## 2. Methods

### 2.1. Study Design and Participants

This study is a secondary analysis of a previously published randomized controlled trial evaluating immune-guided precision care [25]. The original trial prospectively enrolled 493 infertile women aged ≤38 years with normal ovarian reserve undergoing IVF or ICSI at a single ART center between October 2015 and February 2023.

The trial protocol was approved by the Institutional Review Board of University Paris Diderot and registered at ClinicalTrials.gov (NCT02262117). The study followed extended CONSORT guidelines and was monitored by an independent Data and Safety Monitoring Board. A protocol amendment implemented in 2017 allowed frozen embryo transfers and up to two oocyte retrieval attempts.

Among the 493 patients initially enrolled, endometrial immune profiling was successfully completed in 484 women. A balanced endometrial immune profile was observed in 106 patients, whereas 378 had endometrial immune dysregulations. Patients with dysregulated profiles were randomized to either conventional care or immune-guided precision care as previously described [25].

### 2.2. Inclusion and Exclusion Criteria

Eligible participants were infertile women scheduled for IVF/ICSI because of tubal infertility, endometriosis, ovulatory disorders, or unexplained infertility following failed intrauterine insemination. Inclusion criteria required age <38 years and normal ovarian reserve defined by anti-Müllerian hormone (AMH) > 1.5 ng/mL, follicle-stimulating hormone (FSH) < 10 IU/L on day 3, and antral follicle count (AFC) > 6.

Exclusion criteria included severe male factor infertility, uterine malformations, contraindications to immunomodulatory therapies, or planned ART treatment at another center. All participants provided written informed consent before inclusion.

### 2.3. Endometrial Immune Profiling

Endometrial biopsies were performed using a Pipelle catheter during the mid-luteal phase after 7 or 8 days of progesterone exposure in hormonally prepared cycles. In natural cycles, sampling was performed 9 to 10 days after the LH surge or 10 to 11 days after hCG triggering. All biopsies were obtained at least two months before embryo transfer.

As previously described in detail [25], endometrial biopsies were obtained using a Pipelle catheter and divided into two portions. One portion was fixed in 4% buffered formaldehyde for histological dating and CD56 immunohistochemistry, while the second was preserved in RNAlater for molecular analyses. Following histological confirmation of cycle phase, RNA was extracted, reverse-transcribed into cDNA, and quantitative RT-PCR was performed to assess IL-18, IL-15, TWEAK, and Fn14 expression levels. CD56+ uterine natural killer cells were quantified by immunohistochemistry using a standardized automated staining procedure. Endometrial immune profiles were established using a predefined algorithm integrating the IL-18/TWEAK ratio, CD56+ cell count, and IL-15/Fn14 ratio, as previously described and validated.

**Figure 1:**
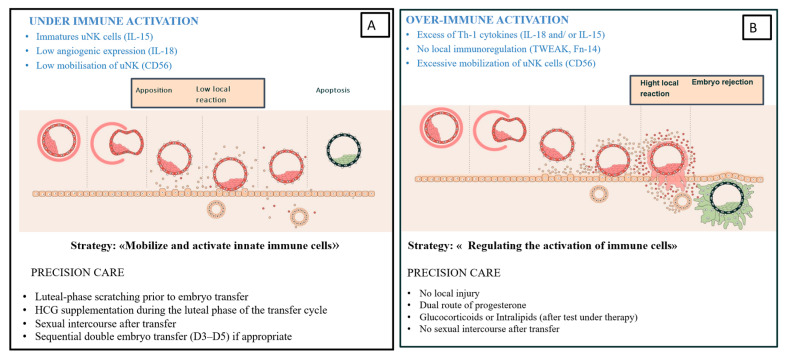
Uterine immune profiling to identify dysregulation and guide precision care.

Based on predefined thresholds derived from fertile controls, immune profiles were categorized as balanced, under-activated, or over-activated. The latter two categories were considered to represent endometrial immune dysregulation (Figure 1). Detailed descriptions of the immune profiling methodology and biomarker validation have been reported previously [23,25]. Patients with diagnosed endometrial immune dysregulation were randomized. Randomization by blocks was made using the electronic server (Cleanweb-APHP) which allocated patients in a 1:1 ratio to the groups “dysregulated—conventional care” or “dysregulated—precision care” once histological and immune results confirmed the mid-luteal phase and the validity of the endometrial immune profile.

### 2.4. Embryo Transfer Procedures and Treatment Strategies

Embryo transfers were performed according to routine clinical practice. Fresh transfers followed controlled ovarian stimulation cycles, whereas frozen embryo transfers were conducted in either natural or hormonally substituted cycles. The interval between endometrial biopsy and embryo transfer did not exceed nine months.

Patients with a balanced immune profile and those randomized to conventional care underwent standard IVF management without immune-targeted interventions. Patients assigned to precision care received individualized treatments aimed at correcting the identified immune imbalance.

#### 2.4.1. Embryo Transfer Policy

Before 2018, embryo transfers were predominantly performed at the cleavage stage. From 2018 onward, prolonged embryo culture to the blastocyst stage and elective single embryo transfer were progressively adopted. Embryo quality and transfer rank were recorded and considered in statistical adjustment.

#### 2.4.2. Immune-Guided Precision Care

Treatment strategies for dysregulated immune profiles were applied according to the previously described protocol [25].

For under-activated profiles (Figure 1A), the objective was to enhance local immune activation and adhesion signaling. Interventions included endometrial scratching [26,27,28], luteal supplementation with chorionic gonadotropin [29,30,31,32], sexual intercourse after embryo transfer [33,34], and in selected cases double sequential embryo transfer [35].

For over-activated profiles (Figure 1B), treatments aimed to reduce excessive immune activation and included glucocorticoids as first-line therapy [36,37,38,39], intralipid infusions as second-line therapy [40,41], and adjusted progesterone support for its immunomodulatory effects [42,43]. Treatment regimens were adapted according to the immune profile under therapy.

### 2.5. Outcomes

The primary outcome was live birth rate per embryo transfer. Secondary outcomes included clinical pregnancy rate, ongoing pregnancy at 12 weeks of gestation, and miscarriage rate. Analyses were conducted according to the modified intention-to-treat principle.

### 2.6. Statistical Analysis

Baseline characteristics were compared between patients undergoing fresh and frozen embryo transfers using appropriate statistical tests depending on variable distribution (chi-square or Fisher’s exact test for categorical variables and *t*-test or Mann–Whitney test for continuous variables).

The primary analysis assessed the interaction between endometrial immune status, treatment group, and embryo transfer type. Logistic regression models were used to estimate odds ratios (OR) with 95% confidence intervals. Absolute risk differences (ARDs) with corresponding 95% confidence intervals were also calculated to provide a measure of absolute effect size. Models included fixed effects for treatment group, embryo transfer type, and a treatment-by-transfer interaction term. Analyses were adjusted for embryo quality and transfer rank.

All statistical tests were two-sided with a significance level of 0.05 and were performed using SAS version 9.4.

## 3. Results

### 3.1. Flow Chart of the Study Population

**Figure 2:**
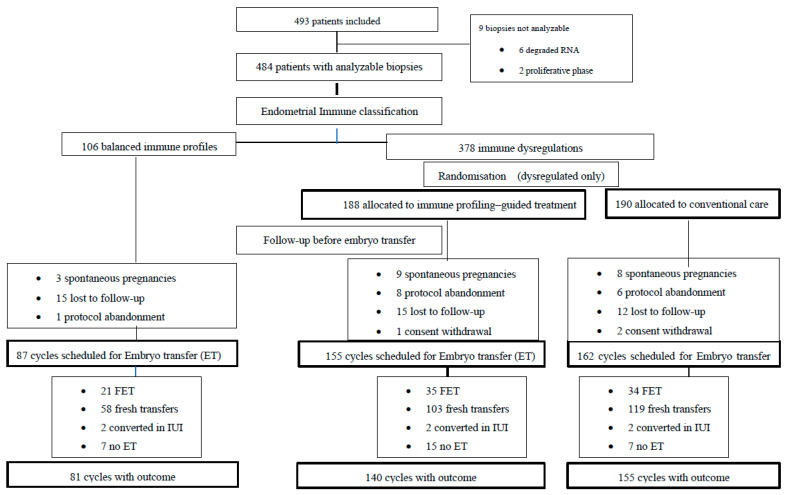
Flow diagram of patient inclusion, randomization and embryo transfer outcomes.

Figure 2 illustrates the flow of patients throughout the study. A total of 493 patients were enrolled between 30 October 2015 and 8 February 2023. Endometrial immune profiling was successfully performed in 484 patients.

Among these patients, 22% (106/484) had a balanced endometrial immune profile, whereas 78% (378/484) presented immune dysregulation. Among dysregulated patients, 190 were randomized to conventional care and 188 to precision care.

Overall, 404 patients were scheduled for embryo transfer (ET), and outcome data were available for 376 patients: 81 with a balanced immune profile, 155 dysregulated patients receiving conventional care, and 140 dysregulated patients receiving precision care.

A total of 286 patients underwent fresh embryo transfer after IVF/ICSI, among whom six cycles were converted to intrauterine insemination (IUI). Ninety patients were scheduled for frozen embryo transfer.

Among fresh embryo transfers, 79.3% were performed using a GnRH antagonist protocol and 20.7% using a long agonist protocol. For frozen embryo transfers, 54% were performed in natural cycles, 7% with mild FSH stimulation, and 39% in hormonally substituted cycles.

### 3.2. Baseline Characteristics of the Study Population

Baseline clinical characteristics according to embryo transfer type (fresh versus frozen) are presented in Table 1.

Frozen embryo transfers were introduced into the protocol in 2018, coinciding with a modification of the embryo transfer policy favoring single blastocyst transfer to reduce multiple pregnancies. Consequently, a higher proportion of blastocyst-stage embryos were transferred in frozen cycles.

A higher proportion of suboptimal-quality embryos were transferred in frozen cycles compared with fresh transfers. In routine practice, the highest-quality embryo is generally selected for fresh transfer, while supernumerary embryos are cryopreserved. Patients undergoing frozen embryo transfer also had a higher frequency of previous implantation failure than those undergoing fresh transfer.

To account for these differences, odds ratios were adjusted for embryo transfer quality and transfer rank.

### 3.3. Endometrial Immune Profiling in Patients Randomized to Conventional Care

Among patients assigned to conventional care, 106 had a balanced endometrial immune profile and 190 had a dysregulated immune profile.

No significant differences were observed between balanced and dysregulated groups with respect to age, previous embryo transfers, type of embryo transfer (fresh or frozen), stimulation protocols, or embryo transfer quality.

Among dysregulated patients randomized to conventional care, 30% had under-activated profiles, 47% had over-activated profiles, and 13.8% had mixed profiles.

### 3.4. Live Birth Rate According to Immune Profile in Fresh and Frozen Embryo Transfer Cycles

To assess the impact of endometrial immune dysregulation on reproductive outcomes according to embryo transfer type, live birth rates were compared between patients with a balanced immune profile (n = 81) and dysregulated patients receiving conventional care (n = 155). No precision care was applied in both groups.

As shown in Table 2, stratified analyses revealed a distinct pattern according to the transfer type. Among patients undergoing frozen embryo transfer, live birth rates were significantly higher in those with a balanced immune profile than in those with immune dysregulation (52.4% vs. 14.7%; adjusted OR 5.32 [1.53–20.92], *p* = 0.008; absolute risk difference +37.7% [8–59]). In contrast, no difference was observed in fresh embryo transfer cycles (34.5% vs. 34.5%; OR 1.09 [0.55–2.18], *p* = 0.79; absolute risk difference 0.0% [−12 to 18]).

### 3.5. Effect of Precision Care According to Embryo Transfer Type

Among dysregulated patients, the effect of precision care compared with conventional management also differed according to embryo transfer type. In frozen embryo transfer cycles, precision care was associated with significantly higher live birth rates than conventional care (48.6% [17/35] vs. 14.7% [5/34]; OR 5.03 [1.66–17.63], *p* = 0.004; absolute risk difference +33.9% [95% CI 11–52]). In contrast, no significant difference was observed in fresh embryo transfer cycles (37.9% vs. 34.5%; OR 1.19 [0.68–2.08], *p* = 0.53; absolute risk difference +3.4% [−8 to 17]).

Consistent with these findings, clinical and ongoing pregnancy rates were higher in dysregulated patients receiving precision care, whereas miscarriage rates per initiated pregnancy were lower but not statistically significant (Table 2).

## 4. Discussion

**Figure 3:**
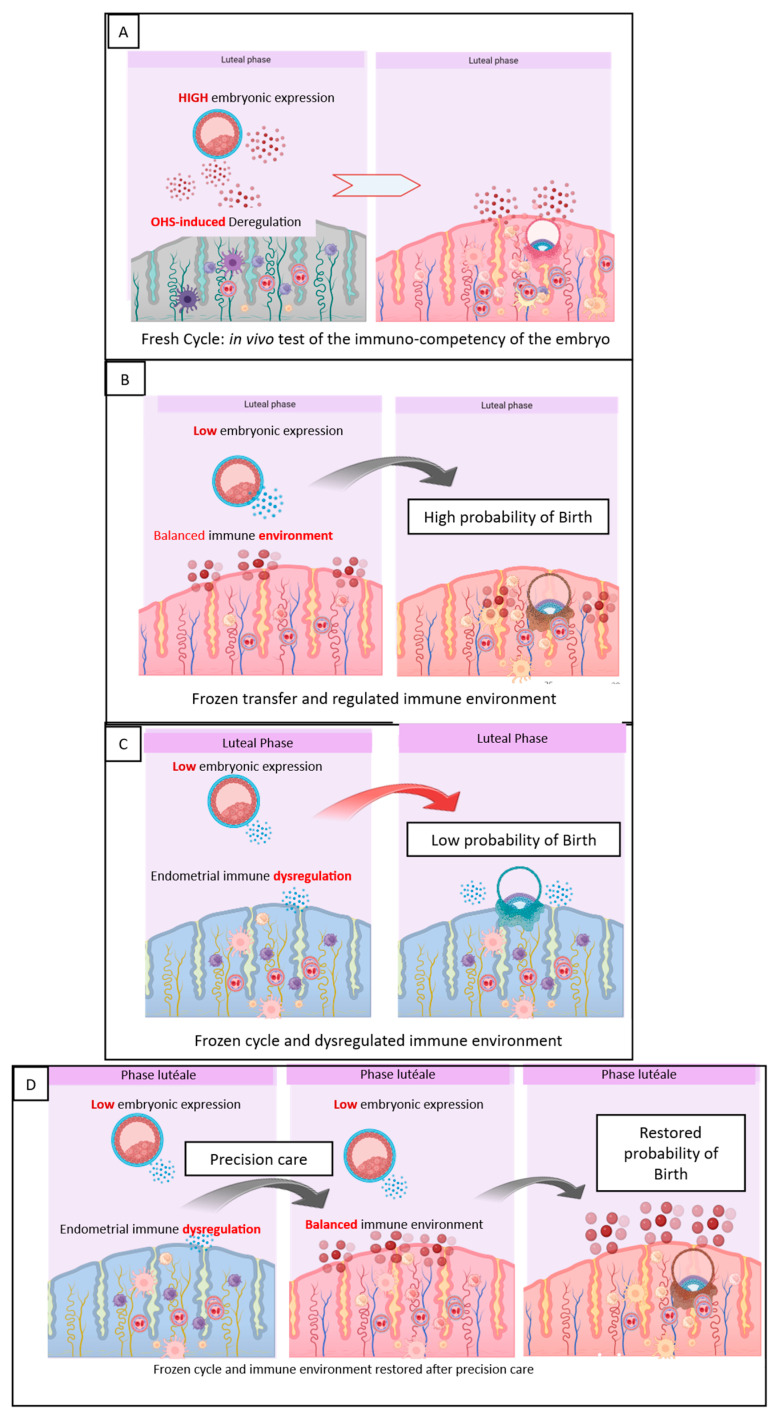
**Endometrial immune environment and embryo–endometrial interactions in fresh and frozen embryo transfer cycles**. (**A**) Fresh embryo transfer in the luteal phase with ovarian hyperstimulation (OHS)-associated endometrial deregulation, including asynchronous decidualization and impaired uterine natural killer (uNK) cell function. Despite high embryonic signaling, implantation occurs in a suboptimal environment and may reflect an in vivo test of embryonic immunomodulatory capacity. (**B**) Frozen embryo transfer in a balanced endometrial immune environment. Adequate receptivity supports implantation despite low embryonic signaling, with a high likelihood of live birth. (**C**) Frozen embryo transfer in an endometrial immune dysregulated state. Low embryonic signaling combined with an unfavorable immune milieu is associated with reduced implantation and low probability of live birth. (**D**) Frozen embryo transfer after personalized intervention restoring endometrial immune balance. Improved embryo–endometrium crosstalk is associated with increased implantation and live birth rates.

To our knowledge, this is the first clinical study specifically examining the interaction between the endometrial immune environment and embryo transfer type in IVF patients. In this prespecified secondary analysis of a well-characterized prospective cohort, we identified a transfer type-dependent effect, predominantly observed in frozen embryo transfer cycles. Endometrial immune status appeared to be strongly associated with implantation success in frozen cycles, where outcomes appeared highly sensitive to immune imbalance. The magnitude of the observed effect (absolute difference > 30%) and its consistency across analyses (balanced versus dysregulated profiles and precision care versus conventional management) support the biological relevance of this association. These findings suggest that endometrial immune competence may represent an important contributor to implantation success rather than a uniform determinant across all embryo transfer settings. Given the limitations of the present analysis, these observations should be considered hypothesis-generating and require confirmation in larger prospective studies. A plausible explanation for the stronger association observed in frozen cycles lies in the distinct endocrine environments of fresh and frozen transfers (Figure 3).

Fresh transfers occur shortly after controlled ovarian hyperstimulation, in a context of supraphysiological steroid exposure and corpus luteum-derived factors known to disrupt endometrial decidualization and impair implantation [44,45]. Beyond endocrine effects, ovarian stimulation may directly alter endometrial immune function. In an implantation-on-a-chip model, Kanter et al. showed that uterine natural killer (uNK) cells derived from hormonally stimulated endometrium displayed reduced capacity to support trophoblast invasion, together with transcriptomic alterations in immune trafficking and inflammatory pathways [46]. Successful implantation following fresh transfer may therefore require compensation for stimulation-induced endometrial dysregulation. Consistent with this, epidemiological studies have reported altered placentation and increased obstetric risks following fresh IVF cycles compared with spontaneous conception or frozen transfer, although findings remain heterogeneous and protocol-dependent [15,47,48]. Furthermore, different ovarian stimulation regimens may not exert identical effects on the endometrial immune environment. GnRH antagonist and long GnRH agonist protocols differ in their endocrine profiles and may therefore differentially influence endometrial immune function. Because the present study was not designed to compare stimulation regimens and the number of patients within each subgroup was limited, we were unable to explore these potential differences. Future studies should investigate whether the relationship between endometrial immune status and reproductive outcomes varies according to the ovarian stimulation protocol used.

Conversely, embryonic signaling capacity may also differ between transfer types. Early embryos release extracellular vesicles carrying immunoregulatory signals that contribute to embryo–maternal dialogue [49,50,51] and actively participate in implantation through adhesion and immune modulation pathways [15,52,53]. Soluble factors secreted by human blastocysts achieving live birth have been shown to selectively modulate the endometrial epithelial transcriptome, enhancing cytoskeletal remodeling and immune pathways [54]. Whether cryopreservation affects these signaling mechanisms remains largely unknown and was not investigated in the present study. Nevertheless, experimental studies in animal models suggest that vitrification may alter embryonic physiology, including miRNA expression, mitochondrial activity, apoptotic pathways, and global transcriptomic profiles [55,56,57,58]. Whether these molecular alterations translate into changes in embryo-derived immune signaling or embryo–maternal communication remains to be determined. Future studies combining endometrial immune profiling with analyses of embryo culture supernatants, cytokine secretion, and embryo-derived extracellular vesicles will be required to determine whether vitrification influences embryo–maternal communication and implantation success.

However, embryo selection strategies must also be considered. High-quality embryos are preferentially transferred fresh, while supernumerary embryos are cryopreserved. Embryos used in frozen cycles may therefore differ in developmental competence or signaling capacity.

Taken together, these data support a context-dependent model of implantation.

In fresh transfers, ovarian stimulation disrupts both decidualization and the endometrial immune environment, requiring embryonic compensation for successful implantation (Figure 3A).

In frozen transfers, performed in a more physiological endocrine context, implantation may rely more directly on intrinsic endometrial immune competence, while the relative contribution of embryo-derived signaling remains to be determined.

This framework provides a potential explanation for our findings. Embryos transferred in fresh cycles may better compensate for local immune dysregulation (Figure 3A), whereas frozen embryos may be more vulnerable to variations in the uterine immune environment (Figure 3B,C). If implantation depends on a finely tuned immune dialogue [49], disturbances in endometrial immune balance may exert a stronger effect when embryonic compensatory capacity is limited.

We therefore hypothesize that precision care targeting the endometrial immune environment before transfer may restore physiological balance and improve implantation outcomes in frozen embryo transfer cycles (Figure 3D).

Several limitations should be acknowledged. First, a major limitation of this study is the relatively small number of frozen embryo transfer (FET) cycles included in the analysis. In addition, patients undergoing FET differed from those receiving fresh transfers, as they more frequently had a history of repeated implantation failure (64.4% with ≥2 previous failed transfers versus 36.1% in the fresh transfer group) and were more likely to receive embryos of lower morphological quality (20.0% top-quality embryos versus 38.2% in fresh transfers). Although all analyses were adjusted for embryo quality and transfer rank, and the main findings remained consistent across analytical approaches, residual confounding related to differences in patient and embryo characteristics cannot be completely excluded. Consequently, the observed associations should be interpreted with caution and considered hypothesis-generating. Confirmation in larger, prospective multicenter studies specifically designed to compare fresh and frozen embryo transfer strategies will be required to validate these findings. Secondly, another limitation is the heterogeneity of endometrial preparation protocols used in frozen embryo transfer cycles. Natural, mildly stimulated, and hormone replacement therapy cycles may differ in their endocrine and immunological environments, potentially influencing implantation and pregnancy outcomes. Because of the limited sample size, particularly in the mild stimulation subgroup, we were unable to perform adequately powered analyses according to FET preparation protocol. Future studies should investigate whether different endometrial preparation regimens modify uterine immune status and its association with reproductive outcomes. Thirdly, another limitation of this study is the imbalance in embryonic developmental stage between fresh and frozen embryo transfer cycles. Blastocysts accounted for the vast majority of frozen embryo transfers, whereas a higher proportion of cleavage-stage embryos were transferred in fresh cycles. Although previous analyses restricted to blastocyst transfers on the same cohort supported the relevance of endometrial immune status [59], the potential influence of embryonic developmental stage on embryo-derived signaling and implantation outcomes cannot be completely excluded. Future studies specifically designed to compare cleavage-stage and blastocyst transfers will be required to further investigate this interaction.

Finally, another limitation relates to the fact that the endometrial immune profiling strategy evaluated in this study was developed by the investigators. Although independent retrospective studies conducted by external groups in the United Kingdom and Canada have reported findings supporting the clinical relevance of endometrial immune profiling [60,61], further validation remains necessary. Prospective multicenter studies performed by independent investigators, ideally using blinded assessment procedures, will be required to confirm the reproducibility, robustness, and clinical utility of the proposed immune profiling criteria.

Overall, our findings support a transfer type-dependent interaction between embryonic competence and the uterine immune environment, with a more pronounced effect in frozen embryo transfer cycles, where implantation appears particularly sensitive to endometrial immune status. Future studies integrating both embryonic and endometrial parameters will be essential to refine this model and to determine whether immune profiling of the endometrium can inform embryo transfer strategies.

## 5. Conclusions

The influence of the endometrial immune environment on implantation appears to depend on embryo transfer strategy. While immune dysregulation had limited observable impact in fresh cycles, its association with reproductive outcomes was predominantly observed in frozen embryo transfer cycles. Given the widespread use of frozen embryo transfer, these findings suggest that assessment of endometrial immune competence may be particularly relevant for high-risk patients with recurrent frozen embryo implantation failures. If confirmed in larger independent studies, this approach could contribute to a more integrated embryo transfer strategy that considers both embryonic and endometrial factors to optimize IVF outcomes.

## Figures and Tables

**Table 1:** Summarizes clinical data of patients by Embryo transfer type.

	Total N = 370	Fresh Transfers N = 280	Frozen Transfers N = 90	*p* Value	
Median Age, years (IQR)	33.4 (31.1; 36.1)	33.4 (31.1; 36.2)	33.5 (31.2; 35.6)	0.6193	^4^
Median AMH, ng/mL (IQR)	3.16 (2.29; 4.63) [n = 365]	3.09 (2.23; 4.48) [n = 277]	3.51 (2.39; 5.02) [n = 88]	0.1145	^4^
Median Number of previous oocytes pick-up (IQR)	1 (0; 1)	1 (0; 1)	1 (1; 1)	0.0028	^4^
Median Number of previous embryos transferred (IQR)	1 (0; 2)	1 (0; 2)	2 (1; 3)	<0.0001	^4^
Previous ET failure, three levels—no. (%)				<0.0001	^13^
Two or more transfer failed	159 (43.0%)	101 (36.1%)	58 (64.4%)		
One transfer failed	83 (22.4%)	72 (25.7%)	11 (12.2%)		
No previous ET	128 (34.6%)	107 (38.2%)	21 (23.3%)		
Median Number of embryos transferred (IQR)	1 (1; 2)	1 (1; 2)	1 (1; 1)	0.0003	^4^
Quality of embryo transfer—no. (%)				0.0015	^13^
No Top	245 (66.2%)	173 (61.8%)	72 (80.0%)		
Top	125 (33.8%)	107 (38.2%)	18 (20.0%)		
Stage of embryos at the time of ET—no./total no. (%)				<0.0001	^15^
Day 2/3 (cleavage group)	73/369 (19.8%)	72/280 (25.7%)	1/89 (1.1%)		
Day5/6 (blastocyste stage)	277/369 (75.1%)	191/280 (68.2%)	86/89 (96.6%)		
Double sequential transfer	19/369 (5.1%)	17/280 (6.1%)	2/89 (2.2%)		

^15^ Fisher’s exact test; ^13^ Pearson’s chi-square test; ^4^ Wilcoxon-Mann–Whitney test; Data are mean (SD), n (%), or median (IQR). Where not all patients had available data, data are shown as n/N (%), mean (SD) or median (IQR) [number of patients with available data].

**Table 2:** Outcomes stratified by treatment and embryo transfer type.

OUTCOME		FROZEN TRANSFER		FRESH TRANSFER			
	No Deregulation n = 21	Precision Care n = 35	Conventional Care n = 34	No Deregulation n = 58	Precision Care n = 103	Conventional Care n = 119	*p* Value ¥ for Interaction
**Live Birth**	11/21 (52.4%)	17/35 (48.6%)	5/34 (14.7%)	20/58 (34.5%)	39/103 (37.9%)	41/119 (34.5%)	
OR (95% CI) *	5.32 (1.53; 20.92)	5.03 (1.66; 17.63)	Reference	1.09 (0.55; 2.13)	1.19 (0.68; 2.08)	Reference	0.05
*p* value	*p* = 0.008	*p* = 0.004		*p* = 0.799	*p* = 0.534		
**ARD (95% CI)**	0.34 (0.08; 0.59)	0.32 (0.11; 0.52)	Reference	0.03 (−0.12; 0.18)	0.04 (0.08; 0.17)	Reference	
*p* value	*p* = 0.01	*p* = 0.002		*p* = 0.698	*p* = 0.49		0.03
**Ongoing Pregnancy**	11/21 (52.4%)	17/35 (48.6%)	5/34 (14.7%)	20/58 (34.5%)	39/103 (37.9%)	Reference	
OR (95% CI) *	5.36 (1.54; 21.06)	5.03 (1.66; 17.62)	Reference	1.05 (0.53; 2.04)	1.14 (0.66; 1.99)		0.04
*p* value	*p* = 0.008	*p* = 0.004		*p* = 0.886	*p* = 0.635		
**ARD (95% CI),**	0.34 (0.08; 0.60)	0.32 (0.11; 0.52)	Reference	0.02 (−0.13; 0.17)	0.03 (−0.09; 0.16)	Reference	0.02
*p* value	*p* = 0.01	*p* = 0.002		*p* = 0.796	*p* = 0.60		
**Clinical Pregnancy**	12/21 (57.1%)	19/35 (54,3%)	9/34 (26.5%)	27/58 (46.6%)	50/103 (48.5%)	52/119 (43.7%)	
OR (95% CI) *	2.79 (0.91; 9.01)	2.85 (1.07; 8.00)	Reference	1.25 (0.66; 2.36)	1.25 (0.73; 2.14)	Reference	0.31
*p* value	*p* = 0.08	*p* = 0.041		*p* = 0.502	*p* = 0.411		
**ARD (95% CI)**	0.25 (−0.01; 0.52)	0.24 (0.02; 0.46)	Reference	0.05 (−0.10; 0.21)	0.06 (−0.08; 0.19)	Reference	0.27
*p* value	*p* = 0.064	*p* = 0.029		*p* = 0.495	*p* = 0.403		
**Early miscarriage/Clinical P**	1/12 (8.3%)	2/19 (10.5%)	4/10 (40%)	7/27 (25.9%)	11/50 (22%)	10/52 (19.2%)	
OR (95% CI) *	0.17 (0.01; 1.23)	0.20 (0.03; 1.13)	Reference	1.58 (0.52; 4.71)	1.24 (0.48; 3.24)	Reference	0.12
*p* value	*p* = 0.121	*p* = 0.086		*p* = 0.420	*p* = 0.663		
**ARD (95% CI), *p***	−0.32 (−0.67; 0.03)	−0.30 (0.64; 0.05)	Reference	0.08 (−0.12; 0.28)	0.04 (−0.12; 0.20)	Reference	0.14
*p* value	*p* = 0.076	*p* = 0.090		*p* = 0.447	*p* = 0.641		

Values are no/total no (%). CI denotes confidence interval, ET embryo transfer. * Adjusted Odds ratio for quality of ET and range of transfer/Adjusted absolute risk differences for quality of ET and range of transfer. ¥ The *p*-value for OD is from the logistic regression model for testing the interaction between the treatment group and embryo transfer, *p* value for ARD is from generalized linear regression model for testing the interaction between the treatment group and embryo transfer. Values for early miscarriage are expressed as n/N clinical pregnancies.

## Data Availability

De-identified participant data, the study protocol, and statistical analysis code are available from the corresponding author upon reasonable request and subject to institutional approval and applicable data protection regulations.
